# Water column compensation workflow for hyperspectral imaging data

**DOI:** 10.1016/j.mex.2021.101601

**Published:** 2021-12-12

**Authors:** Deep Inamdar, Gillian S.L. Rowan, Margaret Kalacska, J. Pablo Arroyo-Mora

**Affiliations:** a Applied Remote Sensing Laboratory, Department of Geography, McGill University, Montréal, QC H3A 0B9, Canada; b Flight Research Laboratory, National Research Council of Canada, Ottawa, ON K1A 0R6, Canada

**Keywords:** Hyperspectral imaging, Depth invariant index (DII), Principal component analysis (PCA), Water column compensation

## Abstract

Our article describes a data processing workflow for hyperspectral imaging data to compensate for the water column in shallow, *clear* to *moderate* optical water types. We provide a MATLAB script that can be readily used to implement the described workflow. We break down each code segment of this script so that it is more approachable for use and modification by end users and data providers. The workflow initially implements the method for water column compensation described in Lyzenga (1978) and Lyzenga (1981), generating depth invariant indices from spectral band pairs. Given the high dimensionality of hyperspectral imaging data, an overwhelming number of depth invariant indices are generated in the workflow. As such, a correlation based feature selection methodology is applied to remove redundant depth invariant indices. In a post-processing step, a principal component transformation is applied, extracting features that account for a substantial amount of the variance from the non-redundant depth invariant indices while reducing dimensionality. To fully showcase the developed methodology and its potential for extracting bottom type information, we provide an example output of the water column compensation workflow using hyperspectral imaging data collected over the coast of Philpott's Island in Long Sault Parkway provincial park, Ontario, Canada.

• Workflow calculates depth invariant indices for hyperspectral imaging data to compensate for the water column in shallow, *clear* to *moderate* optical water types.
• The applied principal component transformation generates features that account for a substantial amount of the variance from the depth invariant indices while reducing dimensionality.
• The output (both depth invariant index image and principal component image) allows for the analysis of bottom type in shallow, *clear* to *moderate* optical water types.

## Graphical abstract

**Figure:**
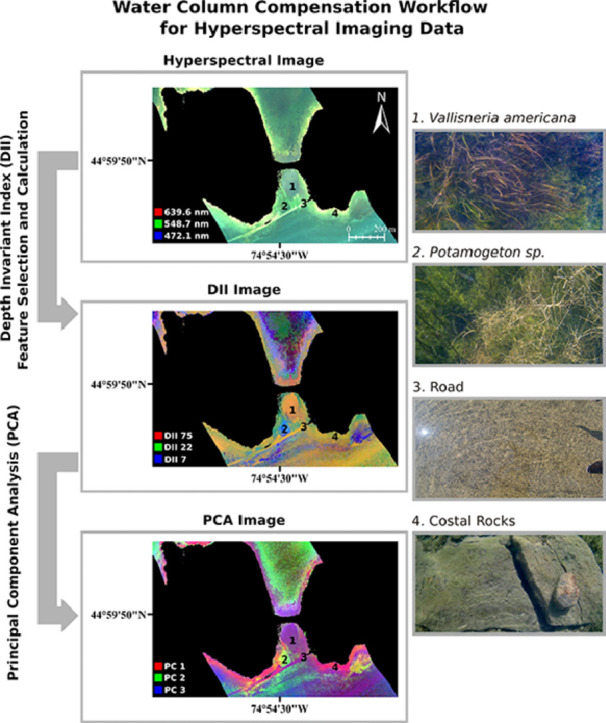

**Specifications table**

**Table:** 

Subject Area:	Environmental Science
More specific subject area:	*Remote Sensing of Optically Shallow Water Aquatic Ecosystems*
Method name:	*Hyperspectral Depth invariant index*
Name and reference of original method:	Lyzenga, D.R. (1978). Passive remote sensing techniques for mapping water depth and bottom features. *Applied Optics, 17*, 379–383 Lyzenga, D.R. (1981). Remote sensing of bottom reflectance and water attenuation parameters in shallow water using aircraft and Landsat data. *International Journal of Remote Sensing, 2*, 71–82
Resource availability:	*Required: MATLAB 2020B with Hyperspectral Imaging Library from Image Processing Toolbox*

*Method details

## Background

Over the past four decades, hyperspectral imaging (HSI) has provided rich spectral-spatial information that has led to a variety of applications for Earth observation [3]. Although most of these efforts have concentrated on analyzing the Earth's land surface, the benthic zones of aquatic ecosystems (both marine and fresh water) have also been successfully studied [2,4,18]. Such applications are the most feasible for optically shallow waters where the reflected electromagnetic energy from the bottom of the water body contributes to the water-leaving signal [10]. Optically shallow waters are typically found in inland and marine coastal environments. In these environments, the waters are optically complex as their light-matter interactions can be highly variable and are primarily influenced by sources such as phytoplankton (Case 1 waters) and mineral particles and dissolved organic matter (Case 2 waters) [11,12]. To extract information about the benthic zones of shallow water ecosystems from reflected electromagnetic radiation, it is critical to compensate for the complex effects of absorption and scattering that occur in the overlying water column [13]. A recently proposed optical water type classification scheme for complex waters (such as those in-land) organizes water types into five categories, from clear to brown, according to their bio-optical properties [21]. Due to the low concentrations of optically significant constituents, it is the most feasible to analyze the benthic zone of aquatic ecosystems with *clear* and *moderate* optical water types using HSI [21].

**Fig. 1:**
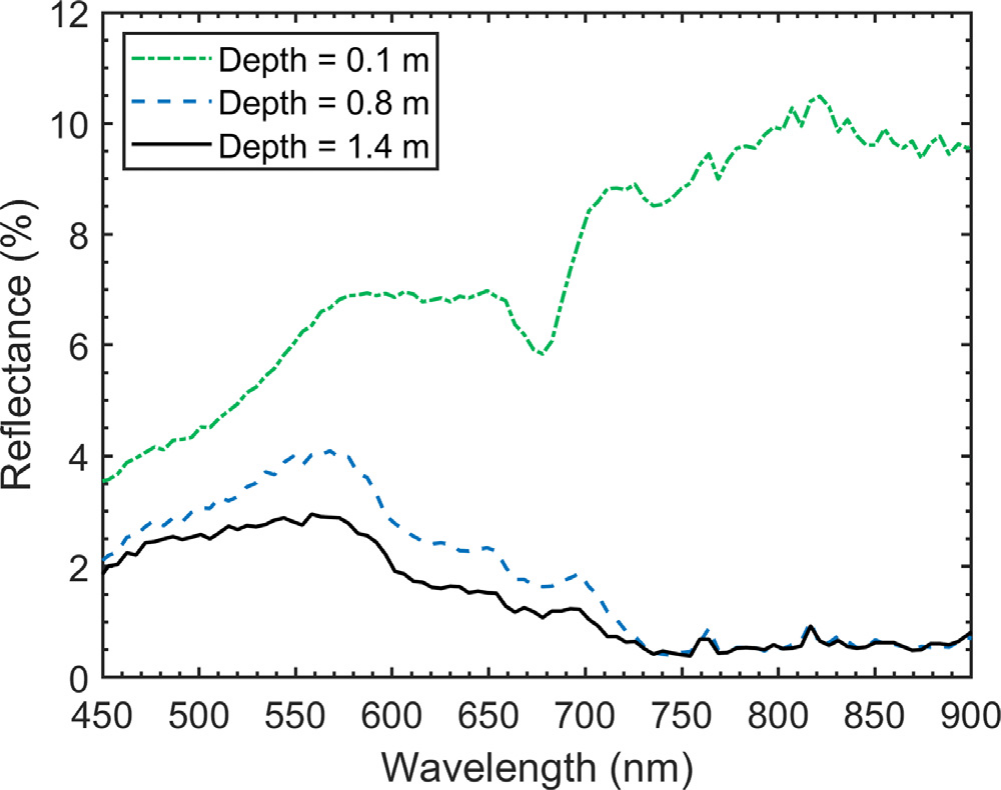
Reflectance spectra from Hyperspectral Imaging data observed over a flooded cement road at the Long Sault Parkway in Ontario, Canada at various water depths. As the water depth increases, the observed reflectance is attenuated.

As electromagnetic radiation traverses the water column in *clear* to *moderate* optical water types, its intensity decreases close to exponentially with increasing depth [7]. The degree to which the intensity of electromagnetic radiation is attenuated varies by wavelength; as the wavelength of electromagnetic radiation increases, more energy is attenuated by the overlying water column. For instance, Gordon and McCluney [5] showed that 90 % of electromagnetic radiation at 600–700 nm is attenuated in high-clarity sea water at a depth of 3.8 m while Green et al. [6] demonstrated that the electromagnetic radiation at 800–1100 nm is completely attenuated at the same depth. The overall effects of the water column can be described by Eq. (1):

(1) $$L_{i}=L_{si}+a\cdot R_{i}\cdot e^{-2k_{i}\cdot z}$$

where $R_{i}$ is the bottom type reflectance at band i that is independent of the water column,$\;L_{i}$ is the radiance observed by the sensor (including the water column) at band i, z is the height of the water column, $k_{i}$ is the water attenuation coefficient (wavelength dependent) at band i, a is a constant (representative of the solar irradiance, the transmittance of the atmosphere and the water surface and the reduction of the radiance due to refraction at the water surface) and $L_{si}$ is the radiance observed over deep water (due to external reflection from the water surface and atmospheric contributions) at band i. Considering Eq. (1), the influence of the water column on the signal recorded by a sensor can create confusion when analyzing bottom type. For instance, the observed reflectance of a substrate at a depth of 0.1 m will look considerably different than the observed reflectance of the same substrate at 1.5 m, especially since the decay rate of electromagnetic radiation is not constant from wavelength to wavelength (see Fig. 1 for an example of reflectance spectra from a submerged cement road at various water depths). In principle, the effects of the water column could be calculated and removed if the water depth and water optical properties were known throughout the scene. However, this is not practically feasible since water depth [7] and optical properties [21] are highly variable in space.

In Wezernak and Lyzenga [23], the variable effects of water column were compensated by calculating the ratio between two spectral bands with attenuation coefficients that were assumed to be equal. Although showing some success, this water column compensation (WCC) methodology was limited as substrates with similarly shaped reflectance spectra (e.g., mud, sand) have nearly equal reflectance ratios [7]. Lyzenga [7] expanded on the simple ratio model, developing a WCC methodology that combines the information from pairs of spectral bands to produce depth invariant indices (DII) that are representative of bottom type. This approach does not require the used spectral bands to have identical attenuation coefficients and thus can produce DIIs that are representative of various bottom types. The only parameter required to generate a DII is the ratio of the water attenuation coefficients between the pair of spectral bands used. The ratios of the water attenuation coefficients can be solved following the method described in Lyzenga [8].

The DII calculations from Lyzenga [7] were originally developed for multispectral imaging data (e.g., [1,9,14,15]), however, have since been applied to HSI data (e.g. [16,19,25]) without fundamental changes to the original formulation. For instance, Rowan et al. [19] calculated DIIs from HSI data to accurately detect submerged aquatic vegetation in a freshwater ecosystem. As shown in this work, various modifications are necessary when compensating for the water column in HSI data by calculating DIIs. For instance, an overwhelming number of indices are generated when calculating DIIs from HSI data as there are an abundance of band pair combinations (e.g. a HSI cube with 100 bands would result in 4950 DIIs). For this reason, feature selection and extraction methodologies help to reduce the dimensionality of the dataset to a more manageable size. Given the intricacies in compensating for the water column in HSI data, an open access WCC tool would help end users to apply HSI data over shallow, *clear* to *moderate* optical water types.

Our work herein describes a data processing workflow that was successfully implemented in Rowan et al. [19] to compensate for the water column in HSI data collected over shallow, *clear* to *moderate* optical water types. Before HSI data can be input into the WCC workflow it must be pre-processed. Given the low signal levels of aquatic environments, conventional radiometric correction processes often result in exceptionally low or negative values of radiance at wavelengths < 450 nm. As such, a two-part in-flight radiometric refinement (IFRR) as described by Soffer et al. [20] is recommended as an initial pre-processing step. Additionally, before being input into the workflow (with or without an IFRR), the HSI data must be atmospherically compensated. In aquatic applications, spectral measurements have contributions from surface reflected radiance (L_SR_), which is incident radiance reflected off the water's surface into the HSI sensor's field of view (e.g. glint). If L_SR_ is being accounted for, L_SR_ removal techniques (see Windle and Silsbe [24] for examples) should also be applied prior to the WCC workflow. Additionally, land pixels must be masked from the imagery.

In the WCC workflow, high frequency noise is first removed using a Savitzky–Golay smoothing filter. After, the effects of the water column are compensated for by calculating a DII from every pair of spectral bands following the methods described in Lyzenga [7] and Lyzenga [8]. A correlation based feature selection methodology is then applied to remove redundant DIIs. In an additional post-processing step, a principal component transformation is applied, extracting informative features from the DIIs while further reducing dimensionality. In this work, we provide a MATLAB script that can be readily used to implement the described workflow, breaking down each code segment so that it is more approachable for use and modification by end users and data providers. To showcase the effectiveness of the developed methodology, the WCC workflow was applied to HSI data collected over the coast of Philpott's Island in Long Sault Parkway provincial park, Ontario, Canada. In the final section, we briefly discuss the application that the WCC compensation was used for in Rowan et al. [19]. Overall, by modifying traditional WCC methodologies (through the inclusion of the Savitzky–Golay smoothing filter, the correlation based feature selection algorithm and the principal component transformation), HSI data can be effectively applied to study aquatic ecosystems with shallow, *clear* to *moderate* optical water types.

## Method workflow

**Fig. 2:**
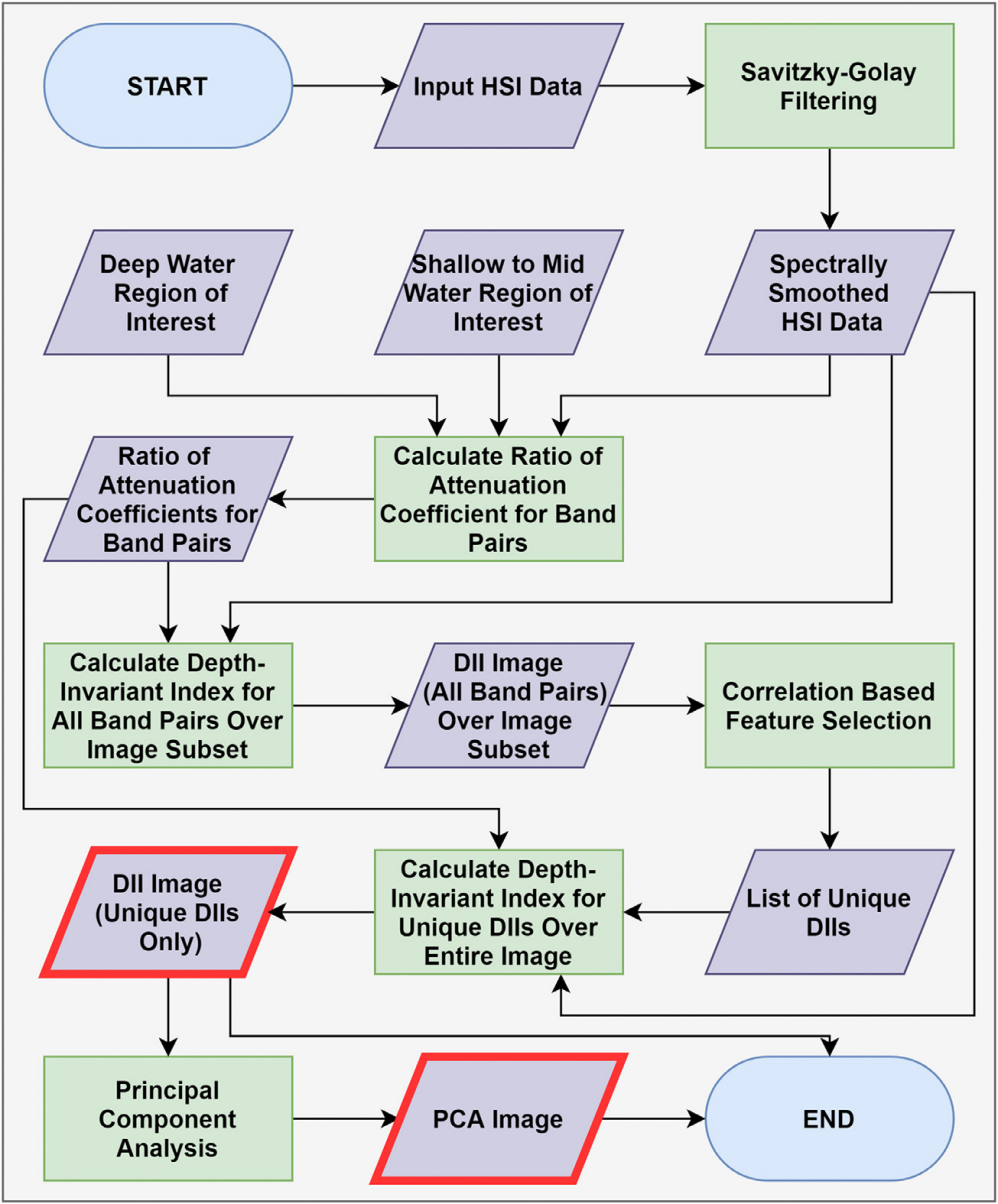
Flow chart of the water column compensation workflow for hyperspectral imaging (HSI) data. The workflow calculates depth invariant indices (DII) for each band pair from the original HSI data. Due to the high dimensionality of HSI data, the number of calculated DIIs are reduced using a correlation based feature selection algorithm. The data dimensionality is additionally reduced using a principal component transformation (PCA). The DII and PCA end products are highlighted with a red boarder in the workflow.

The WCC workflow for HSI data is shown in Fig. 2. It is important to note that the input HSI data to the WCC must be accurately radiometrically corrected, preferably with an IFRR [20], atmospherically compensated and L_SR_ removed (if necessary). Additionally, land pixels must be masked from the imagery.

Even after proper pre-processing methodologies have been applied to HSI data over *clear* to *moderate* optical water types, the water-leaving signal tends to be exceptionally low in comparison to terrestrial imagery [20]. Spectral filtering techniques are often used to remove high frequency noise in low signal data [22]. Accordingly, our WCC first applies a Savitzky-Golay filter of a user specified order. Afterwards, the workflow calculates DIIs following Lyzenga [7].

In the DII calculations, the relationship between water column depth and radiance at a given band,i, is first linearized through the following transformation (Eq. (2)):

(2) $$X_{i}=\ln\left(L_{i}-L_{si}\right)$$

where $X_{i}$ is a variable that is linearly dependent on water column depth and is representative of $L_{i}$. In practice, the inputs to Eq. (2) ($L_{i}$ and $L_{si}$) are not in units of radiance, but reflectance as the imagery must be atmospherically compensated prior to the WCC workflow. The *y*-intercept of a bi-plot of $X_{i}$ and $X_{j}$ (the output of Eq. (2) for given band j that is different than band i) is representative of the bottom surface reflectance independent of the water column and thus a DII [7]. To solve for the DII, Eq. (3) is used:

(3) $$DII_{ij}=X_{i}-\frac{k_{i}}{k_{j}}X_{j}$$

where $\frac{k_{i}}{k_{j}}$ is the ratio of attenuation coefficients between band i and j (slope of the bi-plot of $X_{i}$ and $X_{j}$). Although $\frac{k_{i}}{k_{j}}$ can be linearly regressed with training data, the results depend on which bands are chosen as the dependent and independent variables. This problem can be avoided by minimizing the mean square deviation perpendicular to the regression line to calculate the ratio of attenuation coefficients [8] (Eq. (4)):

(4) $$\frac{k_{i}}{k_{j}}=\frac{\sigma_{i}-\sigma_{j}}{2\sigma_{ij}}+\sqrt{{\left(\frac{\sigma_{i}-\sigma_{j}}{2\sigma_{ij}}\right)}^{2}+1}$$

where $\;\sigma_{i}$ is the variance in $X_{i}$, $\sigma_{j}$ is the variance in $X_{j}$ and $\sigma_{ij}$ is the covariance between $X_{i}$ and $X_{j}$. Following Lyzenga [8], $\frac{k_{i}}{k_{j}}$ is calculated from spectra over a constant substrate along a transect of varying water column depths.

Given the high dimensionality of HSI data, a large number of DIIs can be generated as there is an abundance of band pair combinations (e.g., a HSI cube with 100 bands would result in 4950 DIIs). Therefore, it is not computationally feasible to calculate all possible DIIs across a hyperspectral image. As such, dimension reduction through an initial feature selection is required. In this process, DIIs are calculated over a random subset (user specified size) of the imagery for all possible band pairs. A correlation based feature selection algorithm is then applied to determine the most unique DIIs. The feature extraction method operates under the assumption that features with high correlation are more linearly dependent and hence have almost the same effect on the dependent variable during data applications. When the correlation coefficient between two DIIs is above a user specified threshold, one of the DIIs is dropped. By setting a sufficiently strict correlation threshold, it is possible to reduce the DIIs to a manageable number. After applying the correlation based feature selection algorithm, the selected DIIs are calculated over the entire image. This DII image is given as one of the final end products.

In an additional post-processing stage of the workflow, a principal component transformation is applied to the DII image to generate features that account for a substantial portion of the variance from the DIIs while reducing dimensionality. In this transformation, the user specifies the percentage of the total variance that they want to be explained by the components output in the final data product. The PCA image is the other final output of the WCC workflow.

The described WCC workflow is implemented as a single MATLAB function (HSI_WCC.m). In the following section we break down this function, describing the most important code segments.

## MATLAB function

The presented MATLAB function (HSI_WCC.m) carries out seven main tasks: (1) smooth spectra with Savitzky-Golay filter; (2) calculate DIIs on image subset; (3) select unique DIIs through correlation based feature selection; (4) generate DII image of selected indices; (5) reduce dimensionality of DII image with principal component transformation, identifying important principal components; (6) generate PCA image of the identified principal components; and (7) output DII and PCA images as ENVI standard data files. The inputs and outputs to the function are outlined in the function description:



Before starting task 1, the HSI data needs to be imported as a n (number of image rows) by *m* (number of image columns) by b (number of spectral bands) matrix. Since the built in functions in MATLAB (e.g. sgolayfilt, corrcoef, cov, pca, etc.) require a two-dimensional input, the spatial dimensions of the HSI data matrix also needed to be flattened, creating a n*m by b matrix.



After this initial pre-processing, the imported HSI data are spectrally smoothed with a Savitzky-Golay filter, completing task 1.



To calculate the DIIs, the spectra from a user specified deep water region of interest (ROI) need to be extracted. Similarly, the workflow must extract the spectra from a user specified ROI composed of pixels over a constant substrate at varying water column depths.



Next, $L_{si}$ needs to be calculated. In this WCC workflow, $L_{si}$ is approximated with the mean and standard deviation of the spectra from a user specified deep water ROI. Specifically, $L_{si}$ is equal to the mean deep water spectra minus two standard deviations. If this results in any negative values, $L_{si}$ is set to the mean deep water spectra minus one standard deviation. If this still results in negative values, an error appears, and a new deep water ROI must be selected.



With $L_{si}$ calculated, the workflow linearizes the effect of depth on the spectra from the user specified ROI over a constant substrate along a transect of varying water column depths.



After, the transformed spectra are used to calculate $\frac{k_{i}}{k_{j}}$. In this process, we first calculate $a_{ij}=\frac{\sigma_{i}-\sigma_{j}}{2\sigma_{ij}}$ for all possible band combinations. We then use $a_{ij}$ to calculate $\frac{k_{i}}{k_{j}}$.



Using $\frac{k_{i}}{k_{j}}$, DIIs can be calculated on a subset of the HSI data, completing task 2.



Task 3 is completed by applying a custom correlation based feature selection algorithm to the DIIs calculated on a subset of the imagery. The size of this subset is user specified and should be large enough so that the mean and standard deviation of each DII is representative of the site. The feature selection algorithm first calculates the correlation coefficient between all possible DII pairs. After, the script generates a list of potentially redundant DII pairs (correlation coefficient > then user specified threshold). The script goes through this list one DII pair at a time, identifying the first DII in the pair as redundant. Once a DII is identified as redundant, all of the potentially redundant DII pairs that use that identified DII are removed. By repeating this process until there are no longer any potentially redundant DII pairs, a list of redundant DIIs is generated. The end product of the described feature selection is a logical of redundant DIIs, completing task 3.



By removing the $\frac{k_{i}}{k_{j}}$ values associated with redundant DIIs, it is possible to calculate all the unique DIIs across the entire HSI dataset, completing task 4.



To complete task 5, a principal component transformation is applied to the image generated in task 4. The workflow then identifies the number of principal components that explain a user specified percentage of the total variance.



Task 6 is then completed by generating an image from the selected principal components.



The final task is completed by writing out the generated DII and PCA images. The band names of the DII image are given by the wavelengths used to generate each DII. The band names of the PCA image are given by the component number. Nodata pixels are given a value of -10000.



Below, we provide an example MATLAB code that can be used to call the MATLAB function and generate the DII and PCA images. In our implementation, the two ROIs input into the WCC workflow were drawn in ENVI, exported as comma separated value text files and then imported into MATLAB (see example dataset for more details).



## Example dataset

**Fig. 3:**
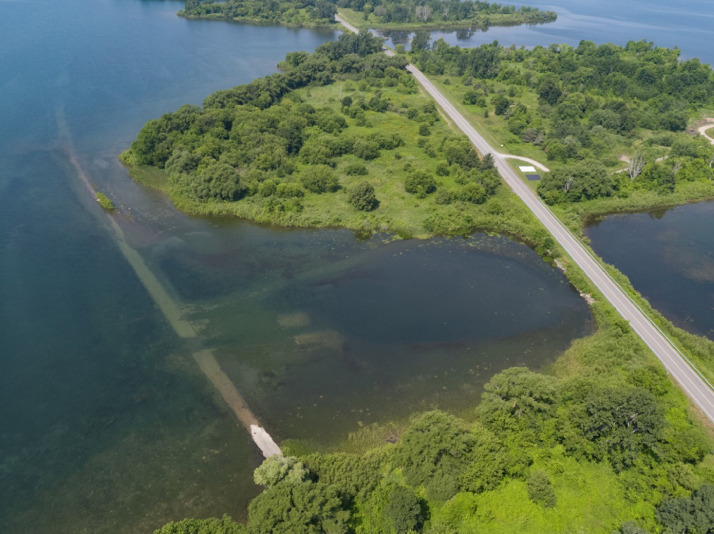
Photograph of Philpott's Island in Long Sault Parkway provincial park. The photograph was captured with the X5s camera aboard a DJI Inspire 2 remotely piloted aerial system.

Here we provide an example output from the WCC workflow applied to HSI data collected over the Long Sault Parkway near Cornwall, Ontario, Canada. The parkway is a connecting group of eleven islands created by flooding the Long Sault rapids during the construction of the St. Lawrence Seaway, which connects the North American Great Lakes to the North Atlantic. The specific example study site is a shallow bay just west of Philpott's Island in Long Sault Parkway provincial park (Fig. 3). The bay has a wide diversity in bottom cover and vegetation.

**Fig. 4:**
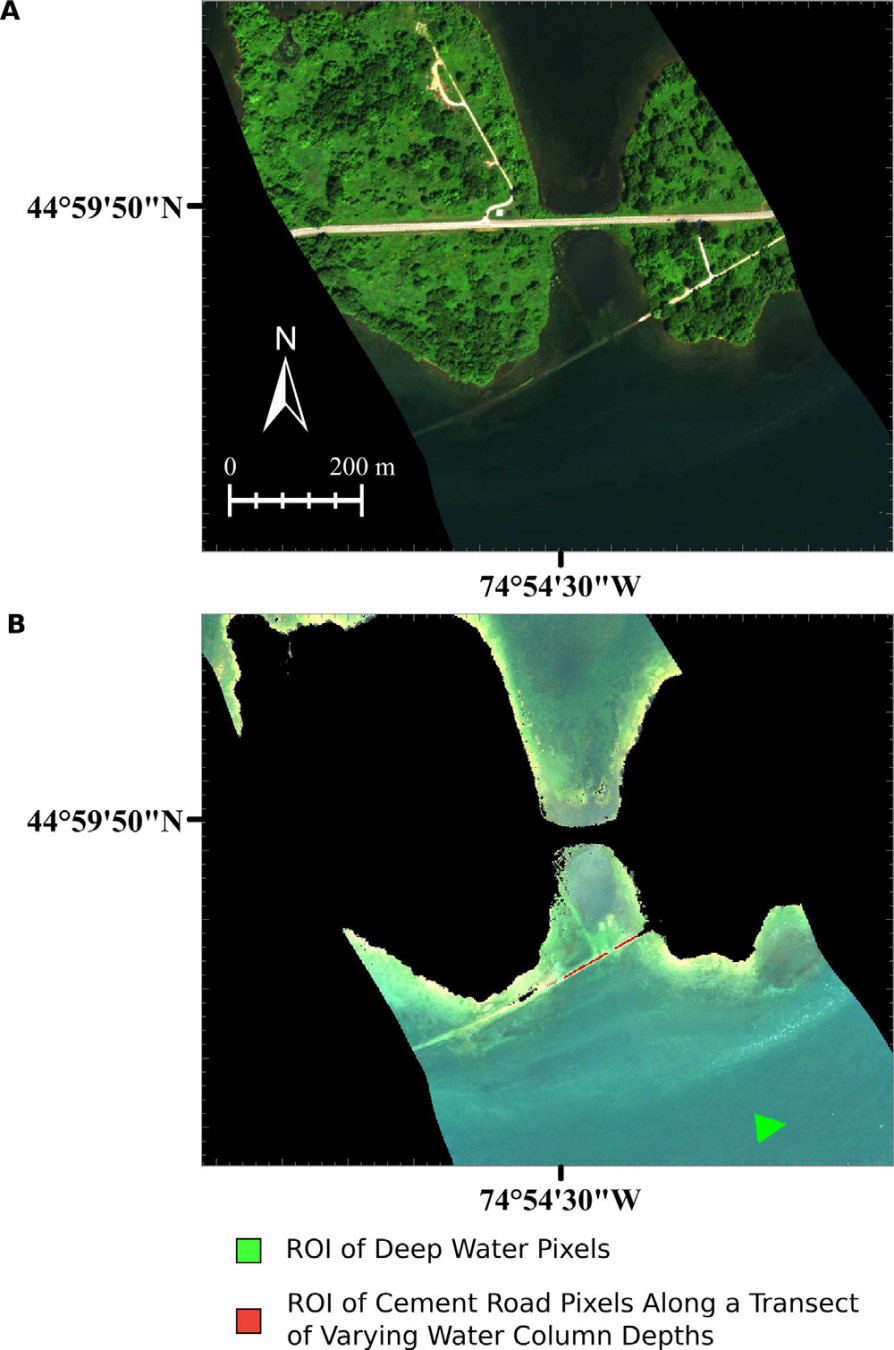
(A) The CASI hyperspectral imaging data collected over the Long Sault Parkway (red = 639.6 nm, green = 548.7 nm, blue = 472.1 nm, linearly stretched between 0 and 13%). Before being input into the water column compensation workflow, the land pixels needed to be masked from the imagery. (B) The CASI hyperspectral imaging data (red = 639.6 nm, green = 548.7 nm, blue = 472.1 nm, linearly stretched between 0 and 3%) after masking out land pixels. The regions of interest (ROIs) required by the workflow are shown in the figure in green (deep water pixels) and red (pixels along a transect of varying water column depths over a constant substrate).

The HSI data used in the WCC workflow (see Fig. 4) was collected by the CASI-1500 hyperspectral imager (ITRES, Calgary, AB, Canada) operated by the National Research Council of Canada – Flight Research Laboratory. The CASI-1500 is a grating-based pushbroom imager with a 39.8° field of view that collects spectral information from 366 to 1053 nm across up to 288 bands with a silicon-based charged coupled device detector. To boost signal levels while capturing spectral information at a sufficiently fine spatial scale (∼1 m) , the spectral bands were summed by a factor of 2 on-chip, resulting in imagery with 144 spectral bands. The imagery was radiometrically corrected with an IFRR applied, atmospherically compensated and geometrically corrected with a standard nearest neighbor resampling. The radiometric correction was carried out with proprietary software provided by the sensor manufacturer. Due to differences between the observed and anticipated at-sensor radiance, the IFRR (i.e., linear correction for mid to high radiance levels, non-linear correction for low radiance levels) developed in Soffer et al. [20] was implemented. The atmospheric compensation was completed in ATCOR4 v7.3.0 (ReSe Applications GmbH, Wil, Switzerland) [17]. The HSI data did not need to be de-glinted as the flight plan and calm atmospheric conditions resulted in no apparent sun glint in the imagery with reflectance of deep water in the NIR < 1% across the field of view. It is important to note that a L_SR_ removal methodology should be applied if there is sun glint or other substantial water surface reflectance artifacts in the imagery. The geometric correction and spatial resampling steps were carried out by software provided by the sensor manufacturer, resulting in a raster HSI dataset with a spatial resolution of 1.16 m. The input parameters to the WCC workflow are shown in Table 1. The location of the two ROIs input into the WCC workflow are shown in Fig. 4; the green ROI is composed of deep water pixels while the red ROI consists of pixels over a constant substrate (cement road) along a transect of varying water column depths from 0.10 to 1.5 m. These ROIs were drawn in ENVI, exported as text files (see DW_roi.txt and sw_mw.txt in supplemental materials). Spectral bands with wavelengths above 950 nm were excluded from the analysis as they were almost completely attenuated by the water column, even at shallow depths (< 10 cm) [19]. Additionally, spectral bands with wavelengths below 400 nm were removed due to low signal levels. To avoid errors in the DII calculations during log transformation, bands with negative reflectance values were also removed from the analysis. In these bands, negative reflectance values have no physical significance and were retrieved during atmospheric compensation due to low signal-to-noise ratios. With the remaining 106 bands, the WCC workflow generated a total of 5565 DIIs. These DIIs were reduced to 124 using the correlation based feature extraction with a threshold value of 0.9. The remaining 124 DIIs were reduced to 31 features using the principal component analysis, generating components that explained 95% of the data variability.

**Table 1:** Parameters used in the water column compensation workflow applied to the CASI-1500 imagery collected over the Long Sault Parkway.

Water Column Compensation Workflow Parameter	Value
Wavelength Range	400–950 nm
Polynomial order for Savitzky-Golay Smoothing Filter	3
Window Size for Savitzky-Golay Smoothing Filter	11
Number of Pixels used in the Correlation Based Feature selection	10,000
Threshold used in the Correlation Based Feature selection	0.9
Percentage of the Total Variance Explained by the Principal Components Encoded in the PCA Image	95 %

**Fig. 5:**
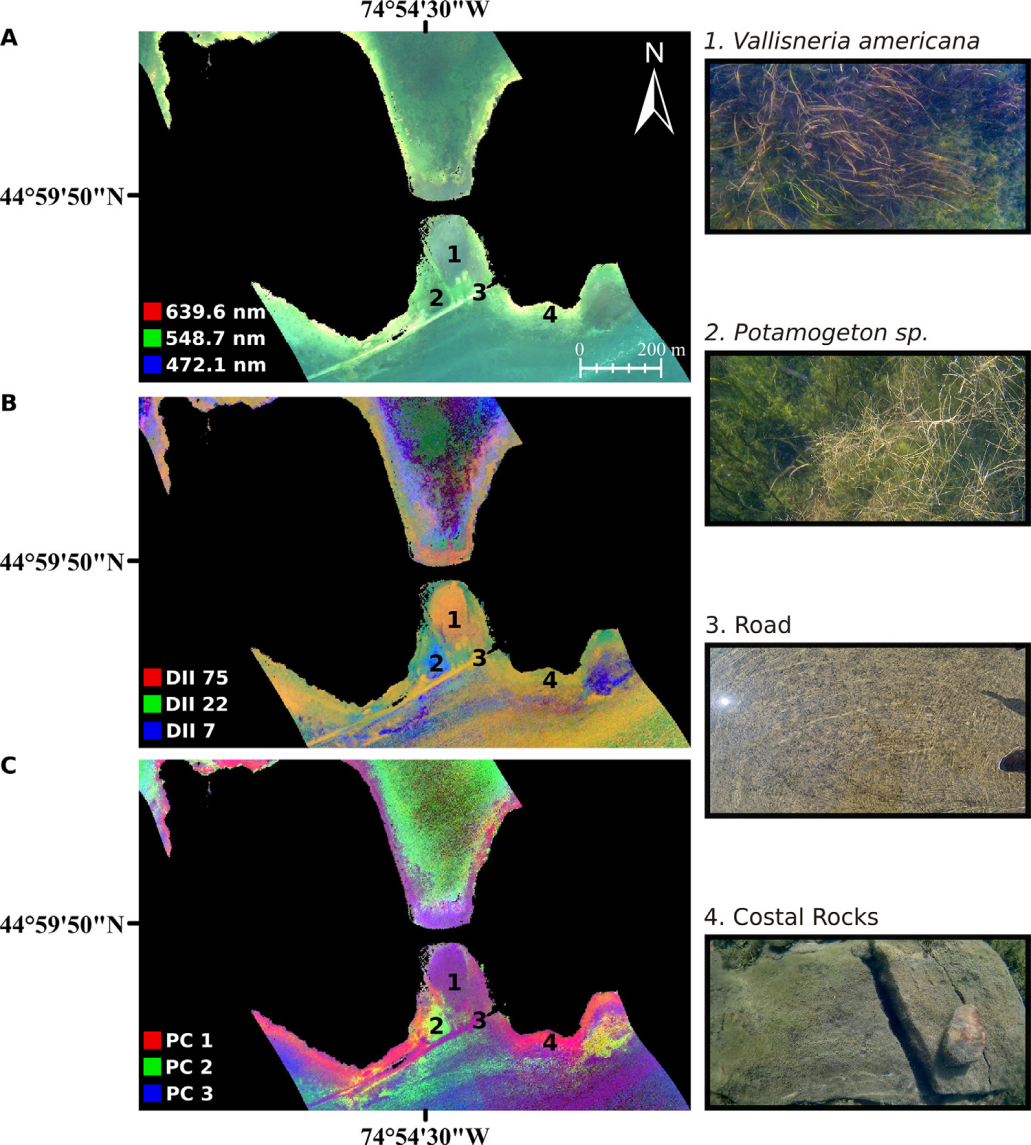
Example output of the water column compensation workflow. (A) Input CASI hyperspectral imagery over Long Sault Parkway (red = 639.6 nm, green = 548.7 nm, blue = 472.1 nm, linearly stretched between 0 and 3%). (B) DII data product (red = DII 75 (682.7 nm & 701.8 nm), green = DII 22 (553.5 nm & 563.1 nm), blue = DII 7 (424.3 nm & 438.7 nm), linearly stretched from minimum to maximum value on extent). (**C**) PCA transformed DII data product (red = PC 1, green = PC 2, blue = PC 3, linearly stretched from minimum to maximum value on extent).

Examples of the DII and PCA images are shown in Fig. 5B and Fig. 5C, respectively. In contrast to the original HSI data shown in Fig. 5A, the DII and PCA images show more variability across the site. For instance, in the true color image, the only easily identifiable shallow water substrates are the cement road and rocky bottom along the edges of each island (Fig. 5A), despite the known diversity in bottom cover at the site. In the DII and PCA images, however, various bottom types are clearly distinguished. In the DII image, dense stands of *Vallisneria americana* and *Potamogeton* sp. are visible in addition to the cement road and rocky substrates (Fig. 5B). The PCA image can similarly be used to detect the variability in bottom cover types (e.g., *V. americana, Potamogeton* sp., rocky substrate seen in Fig. 5C).

**Fig. 6:**
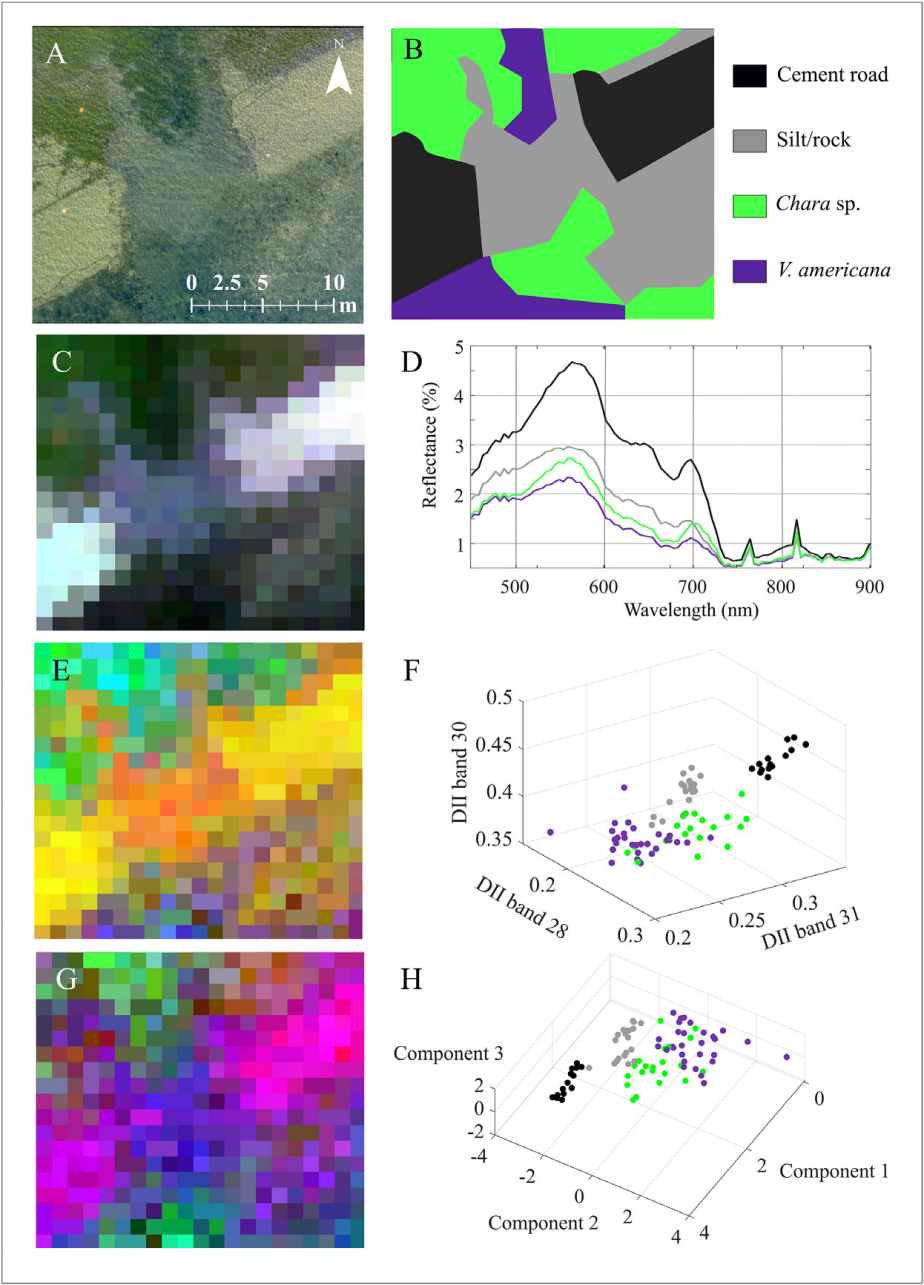
Example application of the water column compensation workflow and resultant class spectra and plots demonstrating the improved performance of the DII and PCA as compared to original hyperspectral imagery. (A) High resolution RGB photograph of the study area (22 m by 22 m region) captured with an X5s camera aboard a DJI Inspire 2 remotely piloted aerial system. (B) Manual delineation of bottom cover classes defined from the orthomosaic and in situ field knowledge of the site. (C) Input CASI hyperspectral image over Long Sault Parkway (red = 644.4 nm, green = 548.7 nm, blue = 472.1 nm, linearly stretched from minimum to maximum value on extent). (D) Plot of mean class spectra extracted from the airborne hyperspectral image. (E) DII data product (red = DII 75 (682.7 nm & 701.8 nm), green = DII 22 (553.5 nm & 563.1 nm), blue = DII 7 (424.3 nm & 438.7 nm), linearly stretched from minimum to maximum value on extent). (F) Plots of the DII values of each class in the study area for DII bands 28 (572.63 nm + 577.42 nm), 30 (596.56 nm + 601.35 nm) and 31 (606.13 nm + 610.92 nm). The DII bands were selected as they had the greatest separability between the two vegetation classes (absolute difference between the mean DII for each class, normalized by the product of the standard deviation of the DII for each class). (G) PCA transformed DII data product (red = PC 1, green = PC 2, blue = PC 3, linearly stretched from minimum to maximum value on extent). (H) Plots of the PCA values of each class in the study area for the first three principal components. The legend in subplot B is applicable to subplots D, F and H.

As an illustration of the WCC workflow effectiveness, points of pure bottom cover were extracted from the untransformed hyperspectral, the DII and the PCA images across a 22 m by 22 m area (Fig. 6). The points were chosen according to manually defined polygons of bottom cover created using a high resolution RGB orthoimage of the area and in situ field knowledge of the site (Fig. 6A,B). The points extracted from the hyperspectral image were plotted across the 450–900 nm region (Fig. 6D). Points from the DII and PCA images were plotted against three axes (Fig. 6F, H). As the reflectance amplitude of aquatic materials depends heavily on the thickness of the overlying water column, first order statistics such as mean reflectance cannot be used to accurately differentiate between classes in the original HSI data. Furthermore, since there is no WCC, constituents within the water column itself are also contributing to the observed reflectance spectra, complicating applications further. The class plots extracted from the DII and PCA images demonstrate that after compensating for the effect of the water column, class clusters are discernable with as few as three dimensions (Fig. 6F, H).

## Effective application of the water column compensation workflow

As previously mentioned, the described WCC workflow was successfully implemented in Rowan et al. [19] to detect submerged aquatic vegetation in the water surrounding the Long Sault Parkway. In particular, a set of target detections was performed on water column compensated HSI data for various classes of interest (i.e., five canopy-forming vegetation types, the paved asphalt road, silt/rock, all vegetation combined, and a non-vegetation class). For pure pixels, the overall recall of the target detection was 87.8% across the individual operational taxonomic units and two non-vegetation classes, and 93.6% for the binary vegetation/non-vegetation classes. In these target detections, submerged aquatic vegetation was detected in 5527 m^2^ of the 66,148 m^2^ of identified water area. Such an approach to monitoring submerged aquatic vegetation could be beneficial in water body management (e.g., prevent the establishment of tall stands of vegetation near water intakes). This application shows how this WCC workflow, and the associated MATLAB script, can easily be used and modified for HSI data applications over shallow, *clear* to *moderate* optical water types.

## Declaration of Competing Interest

The authors declare that they have no known competing financial interests or personal relationships that could have appeared to influence the work reported in this paper.

## Appendix Supplementary materials

MATLAB file: HSI_WCC.m Image, application 1

Deep water region of interest: DW_roi.txt Image, application 2

Cement road region of interest over varying water column depths: sw_mw_roi.txt Image, application 3

Example HSI image subset input to the WCC workflow: 190726_LSP-03B-CA_subset.dat, Image, application 4

190726_LSP-03B-CA_subset.HDR Image, application 5

Example WCC workflow DII image subset: 190726_LSP-03B-CA_DII_subset.dat, Image, application 6

190726_LSP-03B-CA_DII_subset.hdr Image, application 7

Example WCC Workflow PCA image subset: 190726_LSP-03B-CA_PCA_subset.dat, Image, application 8

190726_LSP-03B-CA_PCA_subset.hdr Image, application 9
